# RHOA and PRKCZ control different aspects of cell motility in pancreatic cancer metastatic clones

**DOI:** 10.1186/1476-4598-9-61

**Published:** 2010-03-17

**Authors:** Marco Della Peruta, Cinzia Giagulli, Carlo Laudanna, Aldo Scarpa, Claudio Sorio

**Affiliations:** 1 Department of Pathology, University of Verona, Strada Le Grazie, 8, 37134 Verona, Italy

## Abstract

**Background:**

Our understanding of the mechanism regulating pancreatic cancer metastatic phenotype is limited. We analyzed the role of RHOA and PRKCZ in the motility attitude of two subclones of the pancreatic adenocarcinoma cell line SUIT-2 (S2), with different *in vivo *metastatic potential in nude mice: S2-m with a low metastatic potential and highly metastatic S2-CP9 using RHOA and PRKCZ cell-permeable inhibitory peptides.

**Methods:**

Adhesion assays, cell permeable peptides, RHOA activity assay, western blotting

**Results:**

When used in combination cell-permeable inhibitory peptides partially inhibited cell adhesion by about 50% in clone S2-CP9. In clone S2-m, the effect was limited to 15% inhibition. In a wound healing assay, S2-CP9 was sensitive only to treatment with the combination of both RHOA and PRKCZ inhibitory peptides. Conversely, S2-m was unable to migrate toward both ends of the wound in basal conditions. Migration of cells through a membrane with 8 μm pores was completely abolished in both clones by individual treatment with RHOA and PRKCZ inhibitory peptides.

**Conclusion:**

Herein, we demonstrate a critical role for RHOA and PRKCZ in the regulation of different aspects of cell motility of pancreatic adenocarcinoma and demonstrate the need to inhibit both pathways to obtain a functionally relevant effect in most assays. These results indicate that RHOA and PRKCZ, and their downstream effectors, can represent important pharmacological targets that could potentially control the highly metastatic attitude of PDAC.

## Introduction

Pancreatic ductal adenocarcinoma (PDAC) is the most common type of cancer of the pancreas, accounting for more than 85% of pancreatic malignancies. PDAC is an aggressive and devastating disease characterized by rapid progression and resistance to treatment. With a median survival of less than 6 months, a diagnosis of pancreatic adenocarcinoma carries one of the most dismal prognoses in all of medicine [1].

A key feature of malignant cells is their capacity to invade surrounding tissues and metastasize to distant sites. Little is known about the motile attitude of PDAC cells and the signalling events controlling their motility. These include cell spreading and polarization, as well as generation of focal adhesion, focal contacts, filopodia, lamellipodia, ruffles, and intercellular junctions. Each of these events is under the control of specialized and distinct signaling pathways, which are likely to be altered in cancer cells [2]. In addition, many of the signaling molecules controlling cell motility are also involved in the regulation of cell cycle and cell transformation [3].

The study of cancer cell migration in vitro has been considered a valid model for cancer cell migration as it shares several key features as demonstrated by *in vivo *models and requires GTPase activity which provides a positive feedback mechanism [4].

The process of metastasis involves a complex series of events that include cell transformation and proliferation, vascular invasion at the primary growth site with associated basement membrane degradation, transport through capillary or lymphatic vessels, attachment of tumor cells to endothelial or subendothelial structures at the secondary site, and subsequent growth of a secondary tumor mass.

Analysis of quantitative parameters of cell motility in cancer cells may help to identify intracellular signaling events determining invasion and metastasis. To address some of these issues, we studied two subclones of a cell line derived from a pancreatic adenocarcinoma SUIT-2 (S2) with similar *in vitro *motility but different *in vivo *metastatic potential in nude mice: S2-m, a previously described motile clone isolated in our laboratory [3], and S2-CP9, metastatic to the lung upon subcutaneous implantation [5]. We have previously demonstrated the specific involvement of the PRKCZ isoenzyme in the regulation of pancreatic cancer cell motility [3]. The aim of this study was to extend those findings and understand whether differential regulation of cell motility occurs in the individual clones, by comparing the effects on cell motility and in vitro invasion of RHOA and PRKCZ inhibitory peptides. Instrumental to this are the capability of synthetic peptides, with sequences identical to the endogenous PKC pseudosubstrate region, to inhibit the activity of the different PKC isoforms [6] and the availability of highly selective inhibitory peptides to block RHOA-dependent signaling in a region-selective manner [7-9].

Our results suggest a critical and complementary role of these signaling molecules in the regulation of different aspects of cancer cell movement, and underline the need of combined inhibition of both pathways or a common effector to interfere with cell migration processes in the highly metastatic clone.

## Materials and methods

### Cell lines

S2-m [3] and S2-CP9 [5] subclones of the SUIT-2 PDAC cell line were maintained in RPMI 1640, supplemented with heat-inactivated fetal bovine serum (final concentration 10%) and grown in a humidified 5% CO_2 _atmosphere at 37°C.

### Motility and experimental metastasis assays

Twelve primary tumor samples (Table 1) were dissected after surgery, plated on 24 well plates for short-term culture in RPMI 1640 high-glucose, 10% FBS and 50 μg/ml gentamycin. Motility was evaluated in all samples by time-lapse microscopy exactly as described for S2-m [3]. Cell lines analyzed include MCC1 [10], AVC1[11] and SKPC1[12]. For lung metastasis assay 2 × 10^6 ^S2-m and S2-CP9 cells were resuspended in 0.2 ml of RPMI and inoculated subcutaneously in the flank of four week old *nu/nu *Swiss mice weighing 18-22 g (Charles River, Milan, Italy). The animals were visually inspected every two days for 8 weeks when they were euthanized following the guidelines of the local animal facility. Lungs were stained with India ink through the trachea, fixed in Fekete's solution, and the number of lung colonization was counted under a dissecting microscope following established protocols [13]. Table 2 summarizes the results obtained.

**Table 1:** Motility of pancreatic cancer cells

Primary tumors	Sex	Age	Diagnosis	Motility (μm/min)
PT-1	M	59	PDAC	0,3
PT-2	M	70	PDAC	1 ± 0,2
PT-3	M	61	PDAC	0,4 ± 0,1
PT-4	M	54	PDAC	0,8 ± 0,2
PT-5	F	66	PDAC	0,8 ± 0,2
PT-6	F	76	PDAC	1,5 ± 0,1
PT-7	F	69	PDAC	0
PT-8	M	68	PDAC	0
PT-9	M	70	ACT	0
PT-10	F	63	IPMT	0
PT-11	F	61	PET	0
PT-12	F	42	PET	0
**Cell lines**				
MCC1	/	/	MCT-CR	0
AVC1	/	/	AVC	0,1
SK-PC1	/	/	PDAC	2 ± 0,3
S2-m	/	/	PDAC	3.3 ± 0.8
S2-CP9	/	/	PDAC	4 ± 2

PT = Primary Tumor; PDAC: pancreatic ductal adenocarcinoma, ACT: Acinar Tumor, IPMT: intraductal papillary mucinous tumor, PET: pancreatic endocrine tumor, MCT-CR: mucinous cystic tumor-carcinoma, AVC: ampulla Vateri cancer. Data calculated by evaluation of three microscopic fields containing an average of 10 cells.

**Table 2:** Number of lung metastases in nude mice from subcutaneus implant of the subclones S2-CP9 and S2-m

Mice n.	S2-CP9	S2-m
1	3	0
2	0	0
3	2	3
4	10	0
5	16	0

### Evaluation of RHOA activity and PRKCZ expression

S2-m and S2-CP9 cell lines were serum starved for 24 hours, followed by addition of complete medium for 2 hours at 37°C, 5% CO_2_. Cells were then lysed and RHOA activity was measured by the RHOA G-LISA Activation Assay, colorimetric detection versions, according to the manufacturer's instructions (Cytoskeleton, Inc. distributed by Tebu-Bio, Italy). RHOA activity was evaluated with was measured with a VICTOR X3 Multilabel Plate Reader (PerkinElmer, Shelton, USA) with absorbance set at 490 nm. 10 μg of proteins from the same cell lysates were resolved on SDS-PAGE, electroblotted onto polyvinylidene difluoride membranes (Millipore Corp., Bedford, MA). Non specific binding on the membrane was blocked with 3% bovine serum albumin (BSA, Sigma-Aldrich) in TBS-T buffer (0.05% Tween 20 in Tris-buffered saline pH 7.5) for 1 h at RT. Membrane was incubated with primary antibodies (1 μg/ml) in TBS-T with 1% BSA overnight at 4°C. The antibodies used were anti-PRKCZ (UBI-06473 rabbit polyclonal IgG), anti-RHOA (26C4 SC-418 Mab), anti-alpha-actin (rabbit IgG A2066 from Sigma-Aldrich, Milan, Italy). Blots were washed three times in TBS-T and then incubated for 1 h at room temperature with secondary antibody conjugated to horseradish peroxidase: goat anti-mouse IgG (NA931V, 1:3000, HRP-conjugated from sheep, GE Healthcare, Milan, Italy) and anti-rabbit IgG (NA934V 1:20000, HRP-conjugated from donkey, GE Healthcare) in TBS-T, 1% BSA at RT. Membranes were treated for 30 min at 65°C in 0.5 mM Tris pH 6.7, 2% SDS, 100 mM β-mercaptoethanol and washed before probing with additional antisera.

### RHOA and PRKCZ cell permeable inhibitory peptides

The PRKCZ inhibitory peptide (synthesized at the Stanford University Protein and Nucleic Acid Facility) was dissolved immediately before use at a concentration of 1 mM in DMSO. The peptide sequence from the pseudosubstrate region of human PRKCZ isozyme is SIYRRGARRWRKLYRAN (positions 113-129). The P1-RHOA 23-40 peptide was obtained from the Penetratin-1 (P1) fusion protein expression vector pTm3Hb in which the oligonucleotide encompassing human RHOA bases 67-120 (aa 23-40) was inserted between the BamHI and KpnI cloning sites. Recombinant proteins were expressed in E. coli BL21(DE3)pLysS Gold and purified on heparin columns, dialyzed against PBS, and stored at -80°C. P1-23-40 peptide was synthesized by Sigma-Genosys. A glycine was inserted between P1 and RHOA regions to allow greater flexibility of the peptides. The features and specificity of this tool have been thoroughly described [8,9,14].

### Cell adhesion assay

Adhesion assays were performed. 100 μl of cell suspension (3 × 10^4 ^cells/ml) were seeded on 96-well microtiter plate (Orange laboratories). The day after cells were treated for 18 hours at 37°C with 50 μM P1-RHOA 23-40 peptide. Subsequently, 50 μM of PRKCZ peptide was added and the incubation continued for 2 hours at 37°C. Wells were then washed with PBS, 1 mM Mg^++^, 1 mM Ca^++^, fixed and stained with Crystal Violet (0.75% Crystal Violet; 0.25% NaCl; 1.75% Formaldehyde; 50% Ethanol) for 5 minutes. The plates were then washed twice with MilliRO water to remove excess dye, air dried, and adherent cells lysed by the addition of 100 μl PBS containing 1% SDS. The plate was analyzed in a Packard SpectraCount^® ^Photometric Microplate Reader at 590 nm.

### *In vitro *migration assays

Migration was assessed using transwell inserts with 8 μm pores through a PVP-free polycarbonate membrane filter (Costar Corp., Cambridge, MA). Peptides was added in 600 μl of complete RPMI 10 min before the transwells were transferred to wells, paying attention to avoid air bubbles. 100 μl of cells suspension (1 × 10^5 ^cells/ml) were allowed to migrate for 16 h at 37°C in 5% CO_2_. Non-migrating cells on the upper surface of the membrane were removed by scraping using cotton tipped swabs. Transwells were inserts in a 24 well plate containing 500 μl of Crystal Violet staining solution for 5 minutes, and then treated as previously described.

### *In vitro *wound-healing assay

The *in vitro *wound-healing assay utilized was described by Caveggion *et al. *[15]. Briefly, cells were cultured in 24 well plates until confluent. Peptides were added immediately prior to monolayer scratching with the tip of a pipette in order to wound the monolayer. Photomicrographs at 10× objective magnification were taken after 6 hours thereafter to assess cell migration [16].

### Statistical analysis

We performed one-Way ANOVA analyses with Dunnett's multiple comparison test for the adhesion assay. A p < 0.05 was considered statistically significant.

## Results

### Characterization of S2-m and S2-CP9 clones

We quantified the motility and metastatic capability in nude mice of the SUIT-2 subclones S2-m and S2-CP9. Table 1 describes the values measured in these clones and in other pancreatic cancer cell lines together with a series of primary tumor samples.

The metastatic capability to the lung of S2-m was lower (1 mice out of 5, with 3 metastatic nodules) with respect to S2-CP9 (4 out of 5 mice, with an average of 6 metastatic nodules). Table 2 summarizes the results.

**Figure 1:**
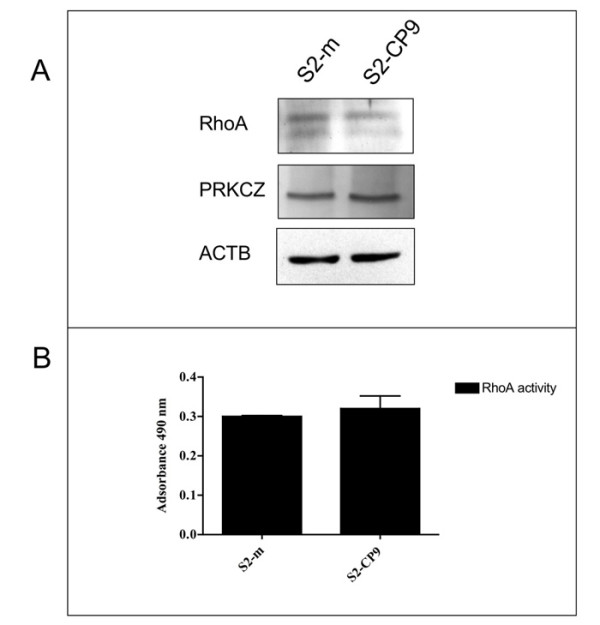
**Expression of RHOA and PRKCZ**. Panel A: The level of expression of RHOA and PRKCZ in S2-m and S2-CP9 subclones of the SUIT-2 pancreas cancer cell line is overlapping. Actin (ACTB) served for normalization. Panel B: RHOA activity assay in S2-m and S2-CP9 clones show that not only expression but also the activity of RHOA is overlapping in both clones (n = 2).

RHOA and PKCZ expression did not differ significantly among clones (Figure 1A). RHOA activity also overlaps among clones (Figure 1B).

### Effects of RHOA and PRKCZ inhibitory peptides on morphology and adhesion

**Figure 2:**
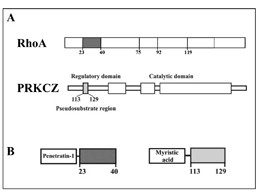
**RHOA and PRKCZ cell permeable inhibitory peptides**. **A**: Site organization of RHOA and PRKCZ showing the effector regions of RHOA (aa 23-40, 75-92 and 92-119), and the inhibitory pseudosubstrate region of PRKCZ (aa 113-129). **B**: Representation of the the plasma membrane translocating peptides. The 23-40 RHOA effector region was fused to Penetratin-1 (left). A myristic acid was added at the N-terminal of the pseudosubstrate region of PRKCZ (right).

**Figure 3:**
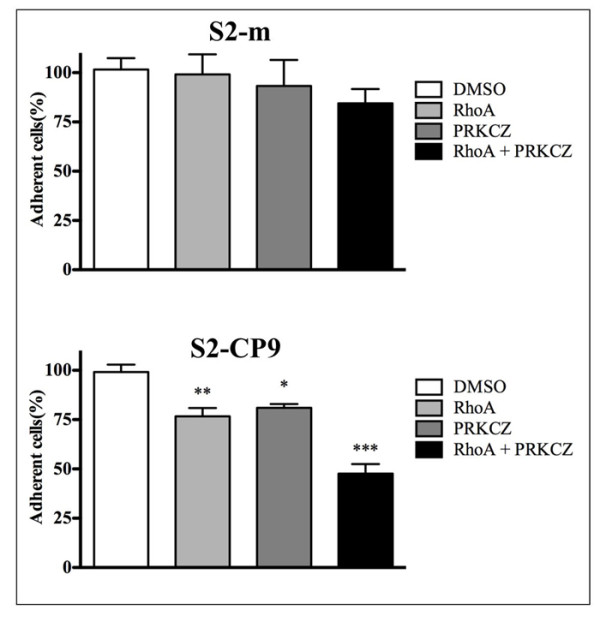
**Role of RHOA and PRKCZ on cell adhesion**. Percentage of the variation of the signal intensity of adherent S2-m and S2-CP9 clones between control and inhibitory peptides-treated cells, with 100% the absorbance found after Crystal Violet staining in the cell lines without treatment. S2-CP9 appear to respond to the individual inhibitory peptides and to their combination while S2-m clone adhesion is unaffected by all the treatments.

We evaluated the adhesion of the SUIT-2 subclones S2-m and S2-CP9. The number of adherent cells after 24 hours in the presence of DMSO or P1 peptide, used as controls, did not differ significantly between S2-m and S2-CP9. Incubation with 50 μM P1-RHOA 23-40 or 50 μM PRKCZ inhibitory peptides (Figure 2) had no affect on adhesion of the S2m cell line when utilized in plate adhesion assays (Figure 3). In contrast, for the S2-CP9 clone, the addition of the individual peptides reduced cell adhesion by 25%. The combination of both peptides induced a marginal inhibition of adhesion in S2-m cell lines, while the inhibition was robust in S2-CP9, where it reached 50%.

### Effects of RHOA and PRKCZ on different aspects of migration ability

**Figure 4:**
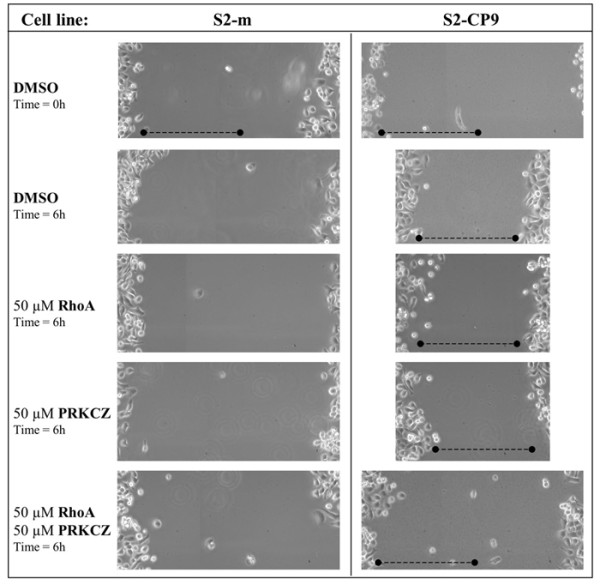
***In vitro *wound-healing assay uncover a selective role for both RHOA and PRKCZ in S2-CP9**. Only the combined effect of 50 μM RHOA and 50 μM PRKCZ peptides (as indicated on the left) are effective in inhibiting wound healing selectively in S2-CP9 clone. S2m appear incapable to cover the wound, demonstrating a defect in random movement, therefore the effect of the inhibition of the indicated pathways can not be evaluated in S2m. Photomicrographs at 10× magnification were taken at the beginning and after 6 hours from the start of treatment to assess cell migration and were mounted together following appropriate reference marks. One representative of 3 individual experiments.

As a result of the different effects of the two peptides observed in the adhesion assay, we evaluated the motility and migration features of the two subclones. Migration ability was measured by wound healing and transmigration assays. The former measures the ability of the cell lines to cover a scratch in a tissue culture plate and is related to the dynamics of cellular movement on a surface. For this purpose, 50 μM RHOA and 50 μM PRKCZ inhibitory peptides were added, alone or in combination, to confluent monolayer of cells immediately prior to scratching with a yellow tip. As shown in Figure 4, 6 hours after wounding, non-stimulated S2-CP9 reached almost confluence (nearly 60% of the wound was covered), whereas RHOA and PRKCZ peptide treated cells remain close to time 0, suggesting a significant reduction of random locomotion. Quantitative analysis, revealed a 90% inhibition of the wound healing by RHOA and PRKCZ in S2-CP9 cells. The S2-m clone was far less efficient in covering the wound, indicating an impairment of random locomotion. Treatment with the peptide was therefore not measurable (see additional file 1).

**Figure 5:**
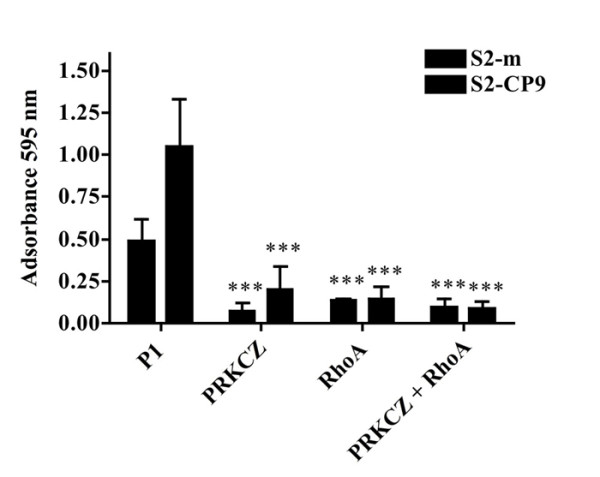
***In vitro *migration assays reveal a role for both RHOA and PKRCZ**. Individual and combined inhibition of PKRCZ and RHOA pathways strongly inhibits the capability of all the clones to migrate through 8 μm pores. OD values determined by Crystal Violet staining of the subclones migrated through the membrane of the Transwell Assay, without and in the presence of the inhibitory peptides indicated. The values of OD are proportional to the number of cells present on the lower surface of the transwell (average values from three individual experiments).

We next performed a transmigration assay, as described in the Materials and Methods, to evaluate the ability of cells to actively modify their shape to pass through 8 μm pores, mimicking the ability of the cells to overstep endothelial cell barriers in micro vessels and to penetrate the extracellular matrix. Treatment with 50 μM PRKCZ or 50 μM P1-RHOA 23-40 inhibitory peptides either alone or in combination almost completely suppressed the ability of both S2-m and S2-CP9 to migrate to the bottom surface of the transwell (Figure 5).

## Discussion

The invasive and metastatic capability of malignancies is associated with the acquisition of anomalous motile behavior by cancer cells, which in turn is dependent on complex biochemical cellular changes whose nature is still not clarified. PDAC is a lethal malignancy whose aggressive behavior depends on several factors, including a strong and early tendency for invasion and metastasis. Even small lesions, less than 1 cm, can be associated with the invasion of both surrounding tissues and lymphatic vessels [17]. In order to address the extent to which the metastatic capability of a cell line is associated to its motility attitude, we investigated two subclones of the same PDAC cell line: a motile clone derived from a clone with a lower metastatic capability in nude mice (S2-m) and a second clone (S2-CP9) that is metastatic to the lung upon subcutaneous implantation. We performed our studies in vitro, and evaluated the sensitivity of the two clones to selective inhibitors of RHOA and PRKCZ. The signaling mechanisms involved in the regulation of PDAC cell motility showed that PRKCZ, an atypical isozyme of the serine-threonine protein kinase C, plays a critical role in maintaining a high motility score in motile subclones. These results were obtained by measuring the effect of specific cell permeable peptides with a sequence corresponding to the pseudosubstrate inhibitory region [3].

PRKCZ is known to associate with Smurf1 to form a complex with Cdc42-PAR6 that induces membrane protrusions and mediates the ubiquitin-dependent degradation of RHOA [18]. PRKCZ was recently shown to act on IKK kinase. IKK members (IKK epsilon) have been involved in F actin assembly, which is necessary for polarized movement in Drosophila [19].

RHOA activity in the cell is primarily related to cytoskeleton regulation. RHO proteins play a central role in regulating cell shape, polarity and locomotion through their effects on actin polymerization, actyomyosin contractility, cell adhesion, and microtubule dynamics [20]. RHOA is a member of the Ras homology family of small GTPases. These proteins cycle from an active (GTP-bound) to an inactive (GDP-bound) conformation by hydrolyzing GTP to GDP. Specific guanine exchange factors (GEFs) reactivate the GTPases by catalyzing the replacement of GDP with a new GTP. Other regulatory factors include GTPase-activating proteins (GAPs), which deactivate RHOA by enhancing its GTPase activity (thus converting the protein more rapidly to its GDP-bound inactive form), and guanine nucleotide dissociation inhibitors (GDIs), which inhibit GAP's functioning and consequently slow the GTPase activity of RHOA [21-23].

Recent studies have shown its indirect involvement (through associated factors) in myosin phosphorylation and cellular responses to stress, such as the formation of focal adhesions and actin stress fibers [24]. It has also been shown to be directly related to myosin chain elongation, actin filament rearrangement, gene expression, cell-shape determination, and cell proliferation [23,24]. These findings and others have sparked recent research interest in the potential involvement of RHOA in oncogenesis. Indeed, over expression of RHOA has been associated with colon, breast, lung, and testicular germ cell cancers as well as in head and neck squamous-cell carcinomas [2]. Different hypotheses regarding RHOA role in these cancers are being explored. One possibility is that the GTPase activity of RHOA provides control for processes necessary for tumorigenesis, such as vesicle transport and cell shape change [2]. Another, not incompatible hypothesis is that metastasis of cancers may be affected by RHOA role in cell motility and process formation [18].

Our experiments demonstrate that both RHOA and PRKCZ are involved in different aspects related to cell adhesion and motility on a surface or through 8 μm pores in S2m and S2-CP9 clones. There are some differences that distinguish the clones: adhesion to plastic plates is only slightly reduced (by 15%) by the simultaneous inhibition of both RHOA and PRKCZ in the S2-m clone. In the highly metastatic clone, however, the same phenomenon is inhibited by 50%. Additionally, as the individual inhibition of RHOA and PRKCZ enzymes leads to a 25% reduction of adhesion, the 50% inhibition obtained by their use in combination suggests an additive effect. These observations imply that distinct, independently regulated signaling pathways, which appear to be partially dependent on RHOA and PRKCZ, regulate cell adhesion in these clones.

When the capability of random movement was tested by the wound-healing assay, S2m was unable to migrate toward both ends of the wound even if its motility overlapped that of S2-CP9. In the latter, only the combined inhibition of RHOA and PRKCZ led to the inhibition of the wound healing capability, a measure of the dynamic of cellular movement on a surface, and individual treatment with the cell permeable peptides was completely ineffective. This indicates that these cells are able to activate an alternative pathway that maintains this capability when the function of one of the enzymes is inhibited.

We next studied how both clones behaved in an experimental setting where migration of the cells occurs through an 8 μm pore membrane, representing a model of cell deformability and movement through tissues. In this case, at variance with the wound healing assay, spontaneous migration through the pores occurs, although to a different extent, in both clones and was almost completely abolished by individual treatment with RHOA and PRKCZ inhibitory peptides. These results validate those obtained in the wound healing assay as they demonstrate that the individual pathways are present and active in both S2-m and S2-CP9 clones This result also suggest that the pathways regulating this phenomenon are either distinct from the ones that regulate adhesion and polarized locomotion or are more sensitive to even a partial inhibition of the signaling originating by RHOA and PRKCZ. Taken together, all these assays indicate that S2-CP9 clone appear to have acquired the capability to better regulate cytoskeleton dynamics compared to clones that are motile but with a lower metastatic capacity. They also suggest that the pathways regulated by RHOA and PRKCZ are connected but capable to independently control specific aspects of cell motility, as shown by the results obtained in adhesion and transmigration assay at variance with that of random locomotion assay.

Protein interaction network analysis reveal interactions between both RHOA and PRKCZ with CDC42, FADD, CASP8 and PRKCA. FADD and CASP8 do not appear to be involved in cellular movement and cytoskeleton rearrangement through actin reorganization [25,26]. We have already shown that PRKCA inhibition is unable to alter cell motility [3].

Together these results highlight the role of CDC42. CDC42 is a small GTPase of the RHO-subfamily, well described as regulator of signaling pathways that controls diverse cellular functions including cell morphology, migration, endocytosis and cell cycle progression. The reorganization of actins into podosomes is controlled by CDC42, a GTP-binding protein [27]. CDC42, as previously described, was found to associate to PRKCZ [28] and acts together with RHOA in the process of depolymerization of actin filaments or microtubules controlling the morphology, motility, and division of most cell types [29].

The common interaction of both RHOA and PRKCZ with CDC42 could explain the experimental results obtained, indicating the resistance to the disruption of the signal originating by the individual partners in the wound healing assay in S2-CP9 clone and the requirement of a combined inhibition of both RHOA and PRKCZ signaling activity to obtain a full biological effect.

Our results indicate that metastatic clones acquire specific capabilities related to cell motility, and that an approach leading to functional interference must take in account that multiple pathways need to be inhibited to reach a functionally relevant effect. In fact, we show that inhibition of a single target or the evaluation of a reduced set of cell motility features can lead to underestimation or even masking the contribution of a selected pathway to the biological phenomenon under study. Our results also suggest RHOA and PRKCZ (and possibly CDC42) represent promising targets for the development of drugs that interfere with the development or progression of the metastatic phenotype and underline the importance of a detailed dissection of the complexity of signaling pathways involved in cancer cell movement.

## Abbreviations and list of keywords

Cancer metastasis, Signal transduction, Cell motility and adhesion, RHO small GTPases, PKC

## Competing interests

The authors declare that they have no competing interests.

## Authors' contributions

MDP carried out the cell biology studies, performed the statistical analysis and drafted the manuscript; CG performed permeable peptides validation experiments and contributed to perform cell biology studies on pancreatic cancer cells; CL designed the peptides, performed motility assays, participated in the design of the study; AS Provided the cellular models and pathological data, critically read the manuscript; CS conceived of the study, participated in its design and coordination, isolated and cultured primary cancers, and finalized the manuscript.

All authors read and approved the final manuscript.

## Supplementary Material

Additional file 1 **Quantification of wound coverage assay of the subclones S2-CP9 and S2-m**. the table show the effect of the indicated peptides on wound healing assay taken at 18 hrs after treatment. Click here for file
