# Supplementary material for: RHOA and PRKCZ control different aspects of cell motility in pancreatic cancer metastatic clones
Source: Mol Cancer. 2010 Mar 17;9:61. doi: 10.1186/1476-4598-9-61 (PMC2846889; doi:10.1186/1476-4598-9-61)
Supplement: Additional file 1 — Quantification of wound coverage assay of the subclones S2-CP9 and S2-m. the table show the effect of the indicated peptides on wound healing assay taken at 18 hrs after treatment. [file 1476-4598-9-61-S1.DOC]

Table S1: Quantification of wound coverage assay of the subclones S2-CP9 and S2-m

|  | **% wound covered at 18 hrs** | |
| --- | --- | --- |
| Peptides | S2-CP9 | S2-m |
| P1 | 80 ± 4 | none |
| RhoA | 70 ± 3 | none |
| PRKCZ | 75 ± 4 | none |
| RhoA + PRKCZ | 10 ± 3 | none |

(n=3 experiments)
